# Aberrant Default Mode Network Underlying the Cognitive Deficits in the Patients With Late-Onset Depression

**DOI:** 10.3389/fnagi.2018.00310

**Published:** 2018-10-03

**Authors:** Xiaoyun Liu, Wenhao Jiang, Yonggui Yuan

**Affiliations:** Department of Psychosomatics and Psychiatry, Zhongda Hospital, Institute of Psychosomatics, Medical School, Southeast University, Nanjing, China

**Keywords:** late-onset depression, Alzheimer’s disease, default mode network, cognitive deficits, genetics

## Abstract

Late-onset depression (LOD) is regarded as a risk factor or a prodrome of Alzheimer’s disease (AD). Moreover, LOD patients with cognitive deficits have the higher risk of subsequent AD. Thus, it is necessary to understand the neural underpinnings of cognitive deficits and its pathological implications in LOD. Consistent findings show that the default mode network (DMN) is an important and potentially useful brain network for the cognitive deficits in LOD patients. In recent years, genetics has been actively researched as a possible risk factor in the pathogenesis of LOD. So, in this review, we discuss the current research progress on the cognitive deficits and DMN in LOD through a combined view of brain network and genetics. We find that different structural and functional impairments of the DMN might be involved in the etiological mechanisms of different cognitive impairments in LOD patients.

## Introduction

Late-onset depression (LOD) is defined as a depression occurring for the first time after the age of 50, 55, 60 or 65 years and the age of 55 is the most commonly used cutoff age (Unützer and Park, 2012; Wang et al., 2016; Geerlings and Gerritsen, 2017). Late-life depression (LLD) contains LOD and early-onset depression (EOD) that recurs or continues into old age. It is a prevalent mental disorder in geriatric population with the prevalence of 8–16% (Blazer, 2003) and its morbidity increases with aging (Büchtemann et al., 2012). For the first antidepressant treatment, the response rate is typically less than 50%, even after multiple treatments, 30 to 40% of the patients with LLD fail to attain full remission (Roose and Schatzberg, 2005; Nelson et al., 2008; Tadayonnejad and Ajilore, 2014).

Late-onset depression (LOD) is associated with a high disability, high recurrence rate, and high family caregiving burden as well as high risk for cognition deficits (Andreescu et al., 2013; Diniz et al., 2014; Kaup et al., 2016). It is also known to be more vulnerable to accelerate brain aging and might predispose to Alzheimer’s disease (AD) by exhausting brain’s structural and functional reserve (Diniz et al., 2015; Freret et al., 2015). LOD patients with poorer cognitive performances exhibit more deficits in a large-scale brain network around AD-related regions and have higher rate of conversion to AD (Wang et al., 2012). Moreover, cognitive dysfunctions have been shown to be persistent in LOD patients even after remission (Yin et al., 2016b). The residual cognitive deficits are believed to be an important risk factor for AD (Alexopoulos et al., 1993; Liu et al., 2018). In a 3-year longitudinal study, dementia eventually occurred in 43% of the elderly depressed patients with cognitive dysfunctions (Alexopoulos et al., 1993). Another study followed up for 5 to 7 years revealed that the conversion rate of dementia in depressed elderly patients with reversible cognitive impairments was 71.4% (Sáez-Fonseca et al., 2007). Butters et al. (2008a) suggested that the combination of mild cognitive impairment (MCI) and depression represented the superimposed AD neuropathology. Moreover, persistent cognitive impairments were found to be associated with the recurrence of LOD (Alexopoulos et al., 2000). Thus, to find specific, objective biomarkers to assist clinicians in improving specific treatments and to establish an individualized diagnosis for the cognitive deficits of LOD has become an urgent problem that needs to be solved.

Imaging genetic studies weight the possible genetic and biological mechanisms behind imaging changes. Recently, genetics has been actively researched as a possible risk factor for the cognitive deficits of LOD (Hou et al., 2010; Wang et al., 2012; Yin et al., 2015b). To improve early identification of LOD which may convert to AD and to achieve further advances in the LOD treatments, we review the progress of neuroimaging and genetic investigations as well as the current clinical status on the cognitive deficits in LOD.

## The Relationship Between LOD and AD

Late-onset depression (LOD) and AD are two common mental disorders that seriously endanger the life and health of the elderly and impose a great burden on our society (Liguori et al., 2018). They may share common mechanisms, such as alterations in glucocorticoid steroids, hippocampal atrophy, inflammatory changes, deficits in brain-derived neurotrophic factors (BDNF), and increased deposition of β-amyloid plaques (Butters et al., 2008b; Byers and Yaffe, 2011; Wang and Dan, 2014).

Alzheimer’s disease is the most common form of dementia in the elderly (McKhann et al., 2011). It is primarily characterized by cognitive deficits and often presents concomitant with depression which may be a reaction of the early cognitive dysfunctions (Espiritu et al., 2001). In turn, depression could lead the accelerated cognitive declines in individuals with pre-existing dementia (Espiritu et al., 2001; Rapp et al., 2011). It was reported that the prevalence of dementia patients who suffer from comorbid depression was from 17 to 50% (Rapp et al., 2011; Leeuwis et al., 2018). A recent study revealed that the occurrence of depressive symptoms in AD might be due to the potential effect of cerebral amyloid angiopathy (Leeuwis et al., 2018).

Along with affective symptoms, a broad range of cognitive deficits have been detected in the acute stage of LOD such as episodic memory, executive function, visual perception function, attention function, working memory, and visual spatial construction function (Hou et al., 2016; Wang et al., 2016). Therefore, when LOD is coupled with cognitive declines, the differential diagnosis between LOD and AD could be challenging. Tsuruoka et al. (2016) have proven that the neurobehavioral cognitive status examination is useful in differentiating LOD and AD although it is just a preliminary study.

The occurrence of depression could even precede the onset of AD (Kobayashi and Kato, 2011). Many studies have shown a relation between a history of depression and risk for AD later in life (Tsolaki et al., 1997; Green et al., 2003; Ownby et al., 2006; Steffens et al., 2011), although a few reported no such risk (Becker et al., 2009). A meta-analysis suggested that depression was a risk factor for AD rather than a prodrome, but it just included studies of AD without including studies of other dementia syndromes (Ownby et al., 2006). A study of 18,726 patients with depressive disorder demonstrated that the rate of dementia tended to increase by 13% with every episode (Kessing and Andersen, 2004). Geerlings et al. (2000) suggested that depression was associated with the increased risk of AD only in subjects with higher levels of education. An 8-year follow-up study revealed that this relationship existed only in men (Fuhrer et al., 2003). However, after controlling for education and sex, a 17-year follow-up study showed that LLD almost doubled the risk of AD (Saczynski et al., 2010).

Some concluded that LOD was a prodrome to AD rather than a risk factor (Li et al., 2011; Barnes et al., 2012). Yeh et al. (2011) believed that LOD was not a risk factor for AD because it had no sufficient duration of the “time-dose effect” on neurotoxicity. Kessing (2012) suggested that EOD may be a risk factor for dementia, whereas LOD may reflect a prodromal phase of dementia.

At present, whether LOD is prodrome or a risk factor of dementia remains controversial. However, no matter LOD is a risk factor for AD or a prodrome, it is generally believed that there is a close relationship between them, especially LOD with cognitive deficits.

## The Characteristic of Cognitive Deficits in LOD

Cognitive impairment was common in depressed older adults, especially in those with LOD. Approximately 50% LOD had cognitive deficits which take various forms (Yeh et al., 2011). Previous findings indicated that the LLD patients with higher levels of education had a greater decrease in memory, executive, and language performances (O’Shea et al., 2015). The cognition deficits in LOD were believed to be an aging-related phenomenon (Yeh et al., 2011). However, Thomas et al. (2009) suggested that this was not caused by aging alone, the illness itself was also accounted for the poorer cognitive performances. In addition, the cognition deficits in LOD were also suggested to be associated with sex and the severity of LOD (Sheline et al., 2006). Vascular lesions and neurotoxicity from the stress-glucocorticoid cascade were two major types of depression-associated pathologies that have been hypothesized to contribute to the cognitive impairments in LLD. It was suggested that vascular lesions mainly resulted in prefrontal-striatal dysfunction, whereas the neurotoxicity from the stress-glucocorticoid cascade mainly led to hippocampal damage (Choi et al., 2017).

Relevant studies of LOD by comprehensive assessment of cognitive domains have demonstrated high rates of impairment in nearly all major cognitive domains (Yin et al., 2015b; Hou et al., 2016; Wang et al., 2016; Choi et al., 2017). Episodic memory and executive function were found to be much worse (Alexopoulos et al., 2005; Hou et al., 2016). A longitudinal study with a mean follow-up of 5.45 years suggested that LLD with cognitive deficits in the domains of memory and executive function during acute stage were the potential predictor for developing dementia (Potter et al., 2013).

After the remission of depressive symptoms, about 45% LLD patients suffered from persistent cognitive deficits; 94% patients who had cognitive dysfunctions remained impaired, while 23% cognitively normal LLD developed cognitive impairments 1 year later (Bhalla et al., 2006). When LOD patients remitted for more than 6 months, they still exhibited poor cognitive performances, besides executive function (Hou et al., 2012). At around 21 months, only executive function and short-term attention function improved significantly in remitted LOD (Jiang et al., 2014; Wang et al., 2015; Yin et al., 2016b; **Table 1**).

**Table 1:** Cognitive deficits in LOD patients compared with healthy aging subjects during different periods.

Cognitive functions	Scale	Acute episodes	Remitted for 6 months	Remitted for 21 months
Overall cognitive performances	MMSE (Katzman et al., 1988)	↓	↓	↓
Auditory episodic memory	AVLT (Barzotti et al., 2004)	↓	↓	↓
Visual episodic memory	CFT (Lezak, 1983)	↓	-	-
Executive function	TMT-A&B (Gordon, 1972)	↓	↑	↑
Visual perception function	SDMT (Wechsler, 1981)	↓	↓	↓
Sustained attention function	SDMT (Wechsler, 1981)	↓	↓	↓
Working memory	SDMT (Wechsler, 1981)	↓	↓	↓
Short-term attention function	DST (Tolor, 1956)	↓	↓	↑
Visual spatial construction function	CDT (Libon et al., 1996)	↓	-	-


*MMSE, Mini-Mental State Examination; AVLT, Audio Verbal Learning Test; CFT, Rey-Osterrieth Complex Figure Test; TMT-A&B, Trail Making Test A&B; SDMT, Symbol Digit Modalities Test; DST, Digit Span Test; CDT, Clock Drawing Test. ↓, represents the decline of cognitive function; ↑, represents the improvement of cognitive function; -, represents there is no data.*

**FIGURE 1:**
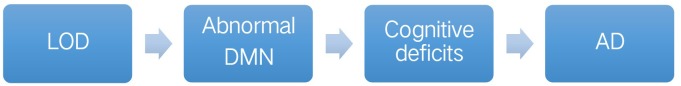
Default mode network (DMN) might be the neural basis for linking LOD with AD. LOD, late-onset depression; DMN, default mode network; AD, Alzheimer’s disease.

Lee et al. (2007) showed that self-reported decline in functional activities was a marker for persistent cognitive impairments in LOD. However, the cognitive deficits in LOD were increasingly considered a disorder of distributed effects of aberrant interactions in the brain (Liu et al., 2018). For decades, morphological and functional magnetic resonance imaging (MRI) have been largely applied to reveal the abnormalities of many different brain regions of LOD, particularly in the default-mode network (DMN) (Hahn et al., 2015; Lebedeva et al., 2015; Hou et al., 2016). DMN might be the neural basis of the connection between LOD and AD (**Figure 1**).

## Default Mode Network Underlying the Cognitive Deficits in LOD

When the “default mode” of brain function was first characterized by Raichle et al. (2001), it was mainly appreciated for the brain remained active in an organized fashion during the resting state. The network was also called “task-negative network” because it exhibited task-induced deactivations (Raichle et al., 2001).

Default mode network (DMN) is generally believed to be a large-scale brain network that encompasses a specific set of brain regions including posterior cingulate cortex/precuneus (PCC/PCu), superior frontal gyrus (SFG), medial prefrontal cortex (mPFC), inferior parietal lobule (IPL), lateral temporal cortex (LTG), angular gyrus (AG), hippocampus and cerebellum (Garza-Villarreal et al., 2015; Hamilton et al., 2015; Mulders et al., 2015). It is often divided into two distinct functional sub-networks, the anterior DMN and the posterior DMN, centers on the ventral mPFC (vmPFC) and the PCC, respectively (Andrews-Hanna et al., 2010). DMN is primarily involved in self-referential functions, such as autobiographical memory, planning the future, remembering the past as well as the perspective taking of the desires, beliefs and intentions of others (Spreng et al., 2009).

The failure to normally down-regulate activity of DMN during an effective reappraisal task was suggested as a biological mechanism of depression (Sheline et al., 2009). Lemogne et al. (2012) demonstrated that rumination in depression might emerge due to a lack of inhibition of DMN. It was reported that anterior to posterior connection within DMN was most severely disrupted with age (Andrews-Hanna et al., 2007). While, in depression, the anterior and posterior DMN showed a dissociation pattern; the functional connectivity (FC) in anterior DMN was increased, decreased in posterior DMN (Zhu et al., 2012; Mulders et al., 2015). The white matter integrity of cingulate bundle which links anterior and posterior DMN has been showed a reduced in LLD (Charlton et al., 2014), that might be the responsible for the dissociation pattern of anterior and posterior DMN.

**FIGURE 2:**
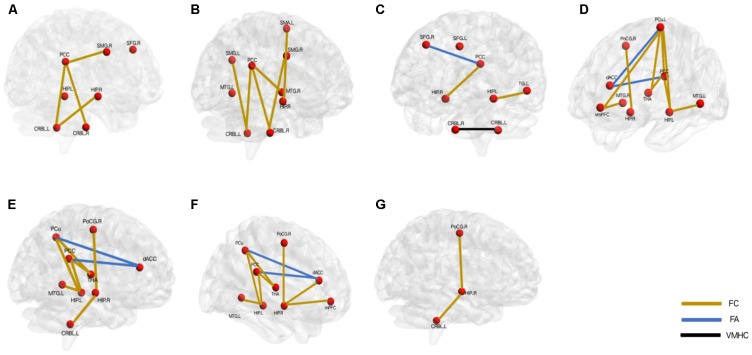
Aberrant default mode network underlying the different cognitive deficits in the patients with late-onset depression. **(A)** overall cognition, **(B)** episodic memory function, **(C)** executive function, **(D)** visual perception function, **(E)** attention function, **(F)** working memory, **(G)** visuo spatial construction function. L, left; R, right; PCC, posterior cingulate cortex; SMG, supramarginal gyrus; SFG, superior frontal gyrus; HIP, hippocampus; CRBL, cerebellum; MTG, middle temporal gyrus; SMA, somatomotor area; dACC, dorsal anterior cingulate; PoCG, postcentral gyrus; THA, thalamus; PCu, precuneus; mPFC, medial prefrontal cortex; vmPFC: ventral mPFC; TG: temporal gyrus. FC, functional connectivity; FA, fractional anisotropy; VMHC, voxel-mirrored homotopic connectivity.

As indicated above, abnormal DMN might be involved in the mechanism of depression. In addition, consistent findings showed that the DMN was an important and potentially useful network for cognitive deficits in the LOD patients (Tadayonnejad and Ajilore, 2014; Hou et al., 2016). The detailed discussion of cognitive impairments and DMN was as the following (**Figure 2**):

### Overall Cognition

Mini-Mental State Examination (MMSE) is a widely used questionnaire for the evaluation of cognitive impairments, especially for AD patients. Generally, MMSE can detect the overall cognitive impairments with sufficient accuracy including memory, language, attention, and orientation function (Pezzotti et al., 2008; Sawyer et al., 2012).

The frontal lobe was believed to be one of the most consistently identified brain regions associated with LOD (Seminowicz et al., 2004). Yue et al. (2015) reported that the abnormal amplitude of low-frequency fluctuation (ALFF) which could directly reflect the intensity of spontaneous neural activity in the right SFG may be related to the overall cognitive dysfunction.

The loss of hippocampal volume was believed to be associated with cognitive deficits specific to AD and was regarded as the most replicable structural abnormalities in LOD (Lloyd et al., 2004; Hickie et al., 2005). Moreover, larger hippocampal volumes were associated with better clinical response to antidepressant treatment (Frodl et al., 2008). It was also suggested that the abnormal hippocampus volume might participate in the dysfunction of overall cognition in LOD patients (Lebedeva et al., 2015). Sawyer et al. (2012) demonstrated that the loss in hippocampal volume could predict the decrease on the MMSE scores over a 4-year follow-up period. An over 2-year longitudinal study also showed consistent results (Steffens et al., 2011).

Previous studies uncovered that BDNF level was negatively correlated with age-related change of hippocampal volume (Erickson et al., 2010). Moreover, the loss of BDNF was suggested play a major role in the pathophysiology of depression (Shimada et al., 2014). LLD patients with MCI showed significantly lower cerebrospinal fluid BDNF levels compared with those without MCI (Diniz et al., 2014). Val66Met (methionine substitution for valine at codon 66), as a common single-nucleotide polymorphism (SNP) in BDNF gene was more frequent in LOD subjects compared with non-depressed older individuals (Lin et al., 2009). It could influence the regulated secretion of BDNF in the hippocampus and was related to lower serum levels of BDNF (Egan et al., 2003). BDNF Val66Met could also increase the risk of AD-related depression and was associated with a better antidepressant response (Zhang et al., 2011). Yin et al. (2015b) found that BDNF Val66Met had an interaction with LOD on decreasing functional connectivity (FC) between the right hippocampi and the left cerebellum and that was associated with the overall cognition dysfunction. Traditionally, the cerebellum was thought to primarily coordinate sensorimotor function and balance. However, recent studies found it was also involved in cognition and emotion which may be due to its extensive anatomically reciprocal connections with the limbic regions and cerebral cortex and could receive projections via the pons from the caudal and rostral anterior cingulate (Stein and Glickstein, 1992; Yin et al., 2015b).

In addition to the abnormal FC between cerebellum and hippocampi, the disrupted FC between cerebellum posterior lobe and PCC was showed to be related with the dysfunctional overall cognition (Yin et al., 2015a). PCC, as the structural and functional core of DMN, had widespread connections with other brain regions. Increasing studies suggested that it was associated with emotion and internally directed cognition such as retrieve autobiographical memory or plan for the future (Gusnard and Raichle, 2001; Hahn et al., 2007; Spreng et al., 2009).

After the follow-up around 21 months, the overall cognition was worse and might be due to the worse FC between PCC and supramarginal gyrus (SMG) (Jiang et al., 2014). SMG is a part of IPL and is involved in language-related function (Gazzaniga et al., 2009). IPL plays an important role in the pathogenesis of MCI and AD (Bai et al., 2008; Del Sole et al., 2008). Two longitudinal studies showed that both the atrophy and reduced regional cerebral blood flow (rCBF) of IPL had high predictive value for the conversion from MCI to AD (Hirao et al., 2005; Karas et al., 2008).

### Episodic Memory Function

Episodic memory involves the ability to encode, retain and recall informations about personal experiences that take place in specific time and place (Budson and Price, 2005). It is suggested as a prodrome of AD (Kidd, 2008) and typically supported by a widespread network of brain regions including PCC, middle temporal gyrus (MTG) and the prefrontal cortex (Cabeza and Nyberg, 2000; Dickerson and Eichenbaum, 2010; Wu et al., 2013). Among MTG structures, the hippocampus has been shown to be critically involved in episodic memory processing (Dickerson and Eichenbaum, 2010). In support of this model, previous studies suggested that the episodic memory decline had a correlation with the abnormal function of PCC and hippocampus in AD patients (Yakushev et al., 2011).

Not only in AD, episodic memory impairment was also showed to be common in LOD (Yuan et al., 2010; Wu et al., 2013; Jiang et al., 2014). Wu et al. (2013) suggested that the disrupted FC between PCC and right MTG was the basis for a decline in episodic memory in the acute stage of LOD. In addition, the impaired visual episodic memory was also suggested to be related to the disrupted FC between PCC and cerebellum posterior lobe (Yin et al., 2015a).

Previous studies suggested the angiotensin-converting enzyme (ACE) gene to be involved in LOD onset (Ancelin et al., 2013), antidepressant response (Baghai et al., 2004) and considered as a candidate gene for AD (Hou et al., 2010). It contained D (deletion) allele and I (insertion) allele. Compared with the ACE ID and II genotypes, the DD genotype was obviously associated with the lowest scores of cognitive performances and was a protective factor for the development of AD (Richard and Amouyel, 2001). Moreover, the ACE-D allele and the status of LOD may synergistically induce larger volume of left MTG and disrupted FC of PCC-left cerebellum, which could increase the risk for visual episodic memory impairment (Hou et al., 2010; Wang et al., 2012).

The human brain is a complex system with small-world architecture. The small-world is an attractive model for it could minimize wiring costs while maximizing the efficiency of information propagation, so it could enable high efficiency in information processing (Sporns and Zwi, 2004). Yin et al. (2016b) revealed that the disturbed small-world properties of the DMN might be a potential biomarker of episodic memory decline. The nodal efficiency likely represented the importance of a nodal region in the whole brain network, and it was suggested that the decreased nodal efficiency of the left putamen was involved in the deficit of episodic memory (Wang et al., 2016).

When LOD patients remitted for more than 6 months, the visual episodic memory improved perhaps because of the increase of left cingulate gyrus volume (Yuan et al., 2008b), the auditory episodic memory improvement might be on account of the increased FC between bilateral SMG and cerebellar (Yin et al., 2015a). In view to both visual and auditory episodic memory, the improvement might owe to the increased FC between right hippocampus and left somatomotor area (Shu et al., 2014).

### Executive Function

Executive function is a primary domain of cognition and involves the abilities of planning and organizing, sequencing, set shifting and response inhibition, these abilities play an important role in goal-directed and complex activities (Alvarez and Emory, 2006). It has been known to be prominently dependent on the incorporation of the prefrontal cortex, parietal cortex, basal ganglia, thalamus, and cerebellum (Rabinovici et al., 2015).

Deficit in executive function is a major contributing factor to the disability of LLD patients (Cahn-Weiner et al., 2007), and a higher degree of executive dysfunction has been linked to poorer or delayed response to antidepressants as well as depression recurrence (Alexopoulos et al., 2005).

Executive function is vulnerable to white matter injury. Fractional anisotropy (FA) is an efficient approach of diffusion tensor imaging (DTI) which could explore the microstructural abnormalities of the white matter tract by measuring the diffusion of water in biological tissue and its reduction represents the destruction of white matter integrity (Yoshiura et al., 2002). Lower FA in distributed cerebral networks in LLD was found to be associated with poor antidepressant response (Alexopoulos et al., 2008). Importantly, the white matter integrity was suggested to be the essential neuronal substrate of cognition function, and Yuan et al.,(2007, 2010) found that the deficit in executive function might be due to the abnormal FA between left posterior cingulate bundles and right SFG. They also showed that the abnormal regional homogeneity (ReHo) of left SFG might be involved in executive dysfunction in LOD patients (Yuan et al., 2008a). ReHo is used to assess the temporal similarity of a given voxel to its neighbors, and the abnormal ReHo is believed possibly to reflect the abnormal activity in the regional brain (Logothetis et al., 2001).

Voxel-mirrored homotopic connectivity (VMHC) is used to indicate the synchrony of spontaneous brain functional activities between symmetrical regions in bilaterally hemispheric architecture. The reduced VMHC of bilateral posterior cerebellar was found significantly associated with executive function changes in LOD patients (Hou et al., 2016). Major depression patients with reduced hippocampal volumes showed more executive dysfunctions (Frodl et al., 2006). Moreover, the FC between left hippocampus and left the temporal cortex could be disrupted by the interaction of LOD and BDNF Met allele in LOD and was responsible for the executive function impairment (Yin et al., 2015b). Recently, Liao et al. (2017) found that the abnormal cerebral blood flow value in calcarine gyrus was also involved in the executive dysfunction.

After treatments, Jiang et al. (2014) suggested that the executive function improve and might be due to the increased FC between right parahippocampal gyrus and PCC.

### Visual Perception Function

Visual perception function refers to the abilities to gather visual information from the environment and to integrate information from experience, motivation and development, that in turn guide our behavior (Butler et al., 2008).

Yin et al. (2015b, 2016a) found that the reduced visual perception function in LOD was correlated with the abnormal FA between PCC/PCu and dorsal anterior cingulate cortex (dACC), the disrupted FC between the PCC/PCu and the thalamus and the changed FC between the right hippocampi and right postcentral gyrus. A recent study provided the risk factors associated with cognitions for LOD patients based on the anterior DMN and posterior DMN and revealed that visual perception function impairment was associated with a lower FC of vmPFC- left PCu and a higher vmPFC- right MTG and PCC- left PCu. The opposite change in the vmPFC-left PCu (anterior DMN) and PCC-left PCu (posterior DMN) might prove the dissociation pattern between anterior and posterior DMN in depression (Liu et al., 2018).

Moreover, the ACE-D allele and the status of LOD might synergistically induce larger volume of left middle temporal gyrus (MTG), which could increase the risk for visual episodic memory impairment (Hou et al., 2010). The interaction of LOD and BDNF Met allele was also responsible for the visual perception dysfunction mainly by decreasing the FC between left hippocampus and left the temporal cortex (Yin et al., 2015b).

After follow-up, LOD patients still demonstrated a poorer visual perception, and Hou et al. (2012) revealed that the poorer function might be due to the decreased volume of right hippocampus. The greater longitudinal deficits in FC between PCC/PCu and the left hippocampus also was found to be correlated with the poorer visual perception function in LOD (Wang et al., 2015).

### Attention Function

Attention is a cognitive process that electively focus on an information while ignoring other perceived information. It contains the sustained and short-term attention, and is also the basis of all other cognitive functions (Rizzo et al., 2000).

Yin et al. (2015b, 2016a) suggested that the reduced sustained and short-term attention function in LOD were correlated with the abnormal FA between PCC/PCu and dACC and the disrupted FC between the right hippocampi and right postcentral gyrus. Meanwhile, BDNF Met allele might have an interaction with LOD and be responsible for the short-term attention and the sustained attention dysfunction through disrupting FC of right hippocampi-left cerebellum and the FC of left hippocampus-the left temporal cortex (Yin et al., 2015b). ACE genetic variants could be involved in the psychopathology and pathophysiology of the sustained attention function in LOD by modulating the microstructural alterations in white matter of left MTG (Yuan et al., 2007).

Late-onset depression patients still demonstrated a poorer sustained and short-term attention function after follow-up that may be due to the greater longitudinal deficit in FC between PCC/PCu and the left hippocampus (Wang et al., 2015). While, in regard to sustained attention function, it might be due to the disrupted FC between the PCC/Pcu and the thalamus (Yin et al., 2016a) and the decreased volume of right hippocampus (Hou et al., 2012).

### Working Memory

Working memory refers to the abilities of temporary storage and manipulation of information in the process of language comprehension, learning and reasoning and is crucial to higher-level tasks such as planning and making (Rabinovici et al., 2015).

Previous study suggested that the reduced working memory function in LOD was correlated with the abnormal FA between PCC/PCu and dACC, the FC between the PCC/Pcu and the thalamus and the FC between the right hippocampi and right postcentral gyrus (Yin et al., 2015b, 2016a).

Genetic studies revealed apolipoprotein E𝜀4 (APOE𝜀4) is associated with increased deposition of amyloid-beta, hyperphosphorylation of tau, as well as impaired neuronal plasticity, so it is regarded as the established genetic risk factor for AD and serves to lower the age of onset (Corder et al., 1993; Liraz et al., 2013). Compared with those lacking the allele, LLD patients who were APOE𝜀4 carriers showed significant suicidality, hippocampal volume reduction, a significantly decreased cognitive function, and a markedly increased risk of dementia (Kim et al., 2002; Hwang et al., 2006; Niti et al., 2009). A study investigated the separate and combined effects of APOE𝜀4 allele and depression on the incidence of dementia in elderly Koreans, and found that LLD patients were at greater risk for incident dementia in subjects with both APOE𝜀4 and depression compared with those without both factors (Kim et al., 2010). It was showed that APOE𝜀4 could increase hippocampal DMN synchronization during rest several years before the clinical manifestation of AD (Westlye et al., 2011). Shu et al. (2014) identified the interactive effect of LOD and APOE𝜀4 on the decreased FC between right hippocampus and bilateral mPFC/ACC, and that involved the working memory dysfunction.

Apart from APOE𝜀4, ACE genetic variants were also demonstrated to be involved in working memory dysfunction in LOD by modulating the microstructural alterations in white matter of left MTG (Hou et al., 2010). BDNF Met allele had an interaction with LOD, and was responsible for the working memory dysfunction mainly by decreasing left hippocampus FC with left the temporal cortex (Yin et al., 2015b).

Late-onset depression (LOD) patients still demonstrated a poorer working memory function after follow-up, which might due to the still decreased volume of right hippocampus (Hou et al., 2012) and the greater longitudinal deficits in FC between PCC/PCu and left the hippocampus (Wang et al., 2015).

### Visuospatial Construction Function

Visuospatial construction function is defined as the ability to see an object as a set of parts and then to construct a replica of the original (Mervis et al., 1999). It was suggested that the impairment of visuospatial construction function in LOD may be due to the increased FC between the right hippocampi and right postcentral gyrus (Yin et al., 2015b) and the decreased FC between the amygdala and the right middle occipital gyrus (Yue et al., 2013).

The interaction of LOD and BDNF Met allele also could be responsible for the visuospatial construction dysfunction by decreasing the FC of the right hippocampi to the left cerebellum (Yin et al., 2015b). In addition, Hou et al. (2010) found that the volume of right ACC modulated by ACE D-allele was also related to visual spatial construction dysfunction.

## Discussion and Prospect

Different structural and functional impairments of the DMN core nodes (PCC, mPFC) and its extension nodes (SFG, PCu, IPL, LTG, AG, hippocampus and cerebellum) might be involved in the etiological mechanisms of different cognitive impairments in LOD patients. The abnormal DMN may have an important early warning value for the conversion of LOD to AD.

Apart from DMN, there are two core neurocognitive networks, the executive control network (ECN) and salience network (SN) which are considered to be relevant contributors to the abnormal cognitive processes observed in LOD (Mulders et al., 2015). ECN includes the lateral prefrontal cortex, the frontal eye fields, the posterior parietal cortex and part of the dorsomedial prefrontal cortex and plays a critical role in cognitive control, working memory, judgment, and decision-making in the context of goal-directed behaviors (Mulders et al., 2015; Li et al., 2017). Alexopoulos et al. (2012) suggested that the decreased FC within the ECN in LLD was predictive of poor treatment response and executive dysfunction. The SN consists of the fronto-insular cortex, the dorsal ACC, temporal poles and the amygdala and is involved in detecting and orienting to both external and internal salient stimuli and events (Menon and Uddin, 2010; Manoliu et al., 2013; Mulders et al., 2015). Disrupted FC within the SN was found might be reflective of disease severity and increased somatization in depression (Paulus and Stein, 2006; Manoliu et al., 2013). Karim et al. (2017) revealed that the FC between ECN and DMN could serve as early markers of treatment response variability in LLD. A recent study revealed that the aberrant ECN-SN connectivity correlated with executive dysfunction in LLD patients (Li et al., 2017).

Converging evidences support brain network dysfunction as a model for the potential neural mechanisms that involved in the impaired cognitive processes in LOD. However, until now, little is known about the altered functional patterns of these three networks. To find potential imaging markers from the FC between DMN, ECN and SN may be a better way to realize image diagnosis, prevention of recurrence and further intervention to prevent LOD progressing to AD in the future study.

Furthermore, potential genetics effect such as APOE, BDNF and ACE behind brain structural and functional alterations were found. However, previous studies were limited to single gene. Candidate gene analyses usually only explain a tiny proportion of brain alterations either structural or functional. Future studies will need to include more genes acting together on brain organized by pathways or through other polygenic analysis. In addition, analyses combing clinical phenotypes, brain imaging and genetics will enhance our understanding of the whole map of cognitive deficits in LOD and AD.

## Author Contributions

YY designed the review and supervised preparation of the manuscript. XL prepared the manuscript. WJ helped with reviewing the manuscript.

## Conflict of Interest Statement

The authors declare that the research was conducted in the absence of any commercial or financial relationships that could be construed as a potential conflict of interest.
